# Case Report: Weakness and Recurrent Falls in an Older Patient

**DOI:** 10.3390/geriatrics10020041

**Published:** 2025-03-13

**Authors:** Mercedes Galloway, Nannette Hoffman, Christopher Lawrence Bray, Ahmed Ebrahim, Brittany Puebla, David Ritchie

**Affiliations:** 1 Graduate Medical Education, College of Medicine, University of Central Florida, 6850 Lake Nona Blvd., Orlando, FL 32827, USAchristopher.bray@hcahealthcare.com (C.L.B.); ahmed.ebrahim@hcahealthcare.com (A.E.); 2 Dr. Kiran C. Patel College of Allopathic Medicine, Nova Southeastern University, 3200 South University Dr., Davie, FL 33328, USA; bp1057@mynsu.nova.edu; 3 General Surgery Residency Program, HCA Florida Kendall Regional, HCA Healthcare, 11750 SW 40th St., Miami, FL 33175, USA; david.ritchie@hcahealthcare.com

**Keywords:** bilateral lower-extremity weakness, falls, geriatric, case reports

## Abstract

**Background/Objectives:** Lower-extremity weakness in older adults is often overlooked, yet it can have reversible or medical causes that contribute to increased falls. Common factors include vision disturbances, impaired balance due to otolith dysfunction, arthritis-related immobility, and lower-extremity neuropathy. This case presents a unique diagnostic challenge in evaluating bilateral lower-extremity weakness and recurrent falls in an older adult, highlighting the complexity of diagnosing conditions with overlapping symptoms. **Case Presentation:** The patient, a woman with a history of a neuroendocrine tumor, experienced progressive weakness in her lower extremities, along with oculomotor and facial muscle involvement, despite extensive testing. Key clinical findings included elevated protein levels in cerebrospinal fluid, suggesting the possibility of an infectious or autoimmune process. A thorough investigation was conducted, including testing for both common and rare conditions such as Guillain–Barré syndrome, Lyme disease, and tuberculosis. **Results:** Despite comprehensive diagnostic efforts, no clear etiology was identified. The patient’s condition was eventually considered to be related to carcinomatosis meningoencephalitis, a rare complication from a previous cancer diagnosis. Given the progressive nature of her symptoms and lack of treatment options, she was transitioned to palliative care. **Conclusions:** This case highlights the importance of a comprehensive differential diagnosis in older patients with unexplained weakness and falls. Rare neurological conditions should not be overlooked, even when more common causes are suspected. Clinicians should remain aware that falls and weakness in older adults may stem from various pathologies, some of which are reversible if identified early, and rare causes must always be considered when standard treatments fail.

## 1. Introduction

Falls in older adults, often associated with bilateral lower-extremity weakness, present a significant diagnostic challenge in clinical practice. A fall is defined as an unexpected and unintentional event where the body descends to a lower surface due to gravitational force [1]. Notably, falls are a leading cause of morbidity and mortality in the elderly population [2]. The causes of lower-extremity weakness are diverse, encompassing neurologic, autoimmune, infectious, and traumatic conditions [2]. Neurological causes such as chronic inflammatory demyelinating polyradiculoneuropathy (CIDP), Guillain–Barré syndrome, and spinal stenosis are well documented, yet differential diagnosis remains complex when multiple overlapping conditions present with similar symptoms.

This case is unique due to the patient’s complex medical history, including a neuroendocrine tumor, alongside various potential contributing factors such as infection and autoimmune disorders. Despite extensive testing, the patient presented with progressive weakness in the lower extremities and oculomotor and facial muscle involvement, which led to a prolonged and uncertain diagnostic journey. Multiple diagnoses, including autoimmune and infectious conditions, were explored, but the rare consideration of carcinomatosis meningoencephalitis, a complication of her previous cancer, added an intriguing dimension to the case. Although a definitive diagnosis could not be confirmed through biopsy, the high clinical suspicion guided her management toward palliative care. In older adults, it is essential to consider not only common causes of weakness, such as age-related sarcopenia and neurodegenerative disorders, but also rarer conditions that might overlap. The failure of conventional treatments, such as physical rehabilitation, emphasizes the need for thorough investigation in cases of recurrent falls. Family and caregiver input can provide critical insights into the patient’s history, helping clinicians to remain open to atypical or rare diagnoses, particularly when patients do not respond to standard treatments.

## 2. Case Presentation

An 82-year-old woman with a significant medical history, including atrial fibrillation, a surgically treated small bowel carcinoid tumor, latent tuberculosis, hypothyroidism, and hypertension, presented to our emergency department from an inpatient rehabilitation facility due to worsening bilateral lower-extremity weakness and frequent falls. Over the past four months, she had been hospitalized three times for similar symptoms, but no definitive diagnosis had been reached. On her fourth admission, her bilateral weakness had progressed to paraparesis, which led to increased concern for both the patient and her husband, as the cause of her symptoms remained undetermined despite multiple hospitalizations. This ongoing uncertainty contributed to significant frustration and anxiety, especially given that her previous hospitalizations had not yielded answers or provided relief. In addition to her worsening lower-extremity weakness, she also experienced visual disturbances and dizziness, further complicating the clinical picture. Both the patient and her husband were understandably distressed and perplexed by the neurological symptoms, which, despite extensive evaluation, continued to lack a clear diagnosis, leaving them uncertain and worried about her ongoing care.

**Figure 1:**
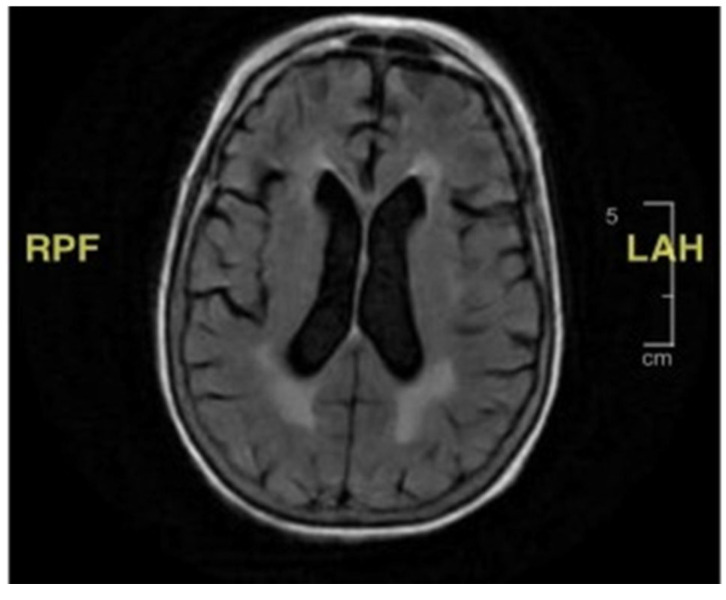
Brain magnetic resonance imaging (MRI). There is no mass effect, hemorrhage, edema, midline shift, or extra cerebral fluid collection. The ventricles and subarachnoid spaces are appropriate in size for the patient’s age.

During the initial hospitalization, she was also evaluated for seven days of diplopia. She had both vertical and horizontal diplopia with mild vertiginous symptoms. Her physical examination then was remarkable for mild right-eye ptosis, subjective diplopia, left-sided facial weakness (left-sided facial droop), and decreased right hemi-body sensation to light touch associated with right lower-extremity weakness. Laboratory studies were non-diagnostic, including thyroid hormone function, vitamin B12, folate, inflammatory markers, and creatine phosphokinase. Radiological imaging, including a cerebral computed tomography scan and magnetic resonance imaging (MRI), showed no acute findings (Figure 1). She was discharged home with ophthalmologist follow-up, and the latter evaluation found no abnormality explaining her symptoms.

**Figure 2:**
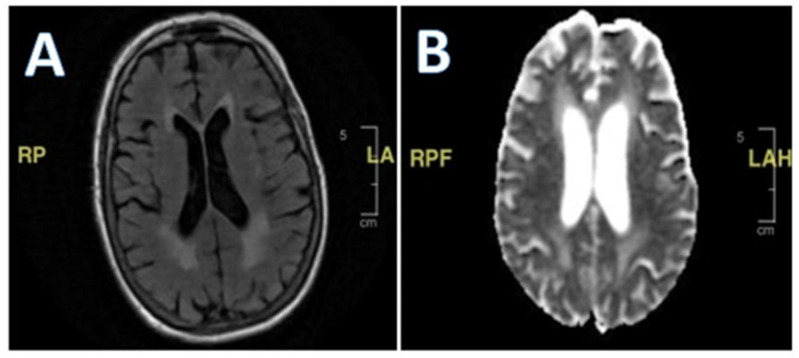
(**A**,**B**) MRI of brain with and without contrast. White matter disease and volume loss indicating chronic microangiopathic changes. No abnormal parenchymal mass or extra-axial collection identified.

**Figure 3:**
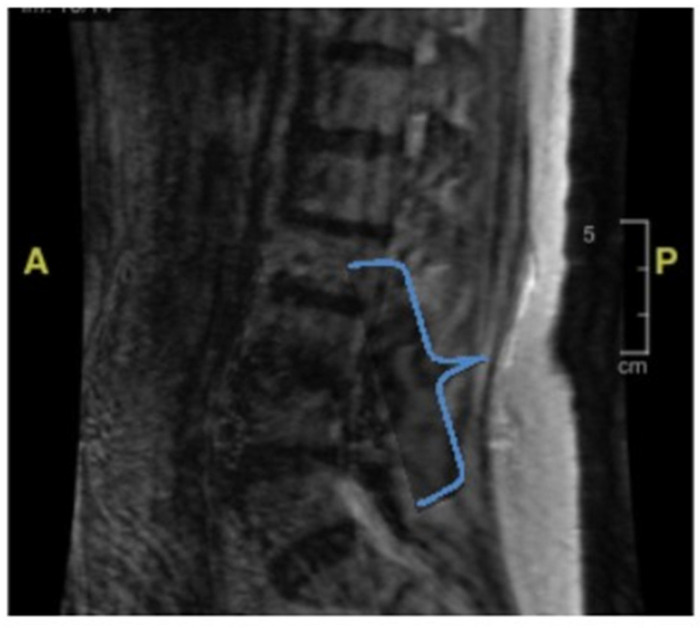
Lumbar spine MRI without contrast. The bracket encloses the area where there appear to be degenerative changes, which are most pronounced at the lumbar vertebral levels of L3–L4 and L4–L5. There is also some degree of spinal canal stenosis, at least moderate at L3–L4. Based upon the MRI, there are no acute fractures or subluxation.

Her second hospital admission was precipitated by repeated ground-level falls from severe bilateral leg weakness. On this occasion, she described worsening back pain with ambulation. She also developed a left facial droop during the second admission. Extensive imaging, including another MRI and CT scan, showed no ischemic changes in the head and neck or acute stroke. Repeat cerebral MRI with and without contrast was negative (Figure 2). Lumbar spine MRI showed advanced degenerative disk disease but no significant pathology explaining her bilateral lower-extremity weakness (Figure 3). Lumbar spine CT showed moderate spinal stenosis at the two lowest vertebrae of the lumbar spine and a 2 mm anterolisthesis. The patient did not exhibit urinary or fecal incontinence.

The patient was admitted for the third time from home following another fall associated with a pre-syncopal episode when rising from a chair. She reported that, during the prior three months, she had experienced frequent falls without head trauma or loss of consciousness. In one 24 h period, she fell five times and required a walker to ambulate. Radiological imaging showed no acute musculoskeletal injury. Upon reviewing the patient’s medication list, all sedating medications were discontinued since the previous admission, yet the frequent falls persisted.

During the third admission, she developed diarrhea with a one-week duration. Stool studies confirmed campylobacteriosis, which was treated with azithromycin. During this hospitalization, the patient once again developed a left-sided facial droop. Extensive imaging, including further head CT and CT angiography (CTA), showed no acute head and neck vascular ischemic changes. There were no spinal MRI findings (Figure 3) that could explain her lower-extremity weakness. During this admission, she underwent her first lumbar puncture, with the results displayed in Table 1. Ultimately, the patient was diagnosed with Bell’s palsy and completed 7 days of prednisone and 5 days of acyclovir. By this point, she had developed an immense fear of falling and was discharged to a rehabilitation facility for mobility and activities of daily living therapy.

The patient presented from the above rehabilitation facility for her fourth admission with bilateral lower-extremity asymmetric motor and sensory deficits. During this admission, her exam showed asymmetric lower-extremity weakness with full strength in her bilateral upper extremities. Her right leg had adequate quadriceps and hamstring strength but poor dorsiflexion. Her left lower extremity showed poor hamstring strength (1/5 in severity) but adequate distal lower-extremity strength. She had weak bilateral hip flexion.

Thus, this patient had persistent, progressively worsening bilateral leg weakness over five months without a definitive diagnosis. During this admission, the lumbar puncture was repeated. The cerebrospinal fluid (CSF) analyses for both lumbar punctures are shown in Table 1. The results were significant for profound hypoglycorrhachia with elevated protein and lymphocytic pleocytosis. The CSF showed no myelin basic protein, oligoclonal banding, or epithelial malignancy. Her spinal fluid culture grew Propionibacterium acnes, for which she received two weeks of ampicillin without improvement. She also had a third lumbar puncture, as seen in Table 1.

Given the CSF findings, there were concerns for a possible fungal or viral infection. She had a positive QuantiFERON-TB Gold (QFT) test and an elevated beta-(1,3)-D-glucan level of 285 (60–79 mg/mL as the indeterminate). However, the CSF polymerase chain reaction (PCR) results for herpes simplex virus (HSV) I and II, varicella, West Nile virus, and tuberculosis were all negative; this was consistent with the results of the previous two separate lumbar punctures, distanced by time post-antibiotics. Culture and staining were negative for fungus, acid-fast bacillus (AFB), and venereal disease research laboratories (VDRL). Cerebrospinal fluid cryptococcus antigen and brucella titers were negative. Full serum infectious diagnostics were all negative or non-reactive, including brucella, coxsackie, and HIV antibodies.

Of note, CSF Lyme antibodies were positive for elevated IgG and IgM levels of 2.12 (reference range 0.00–0.09) and 0.28 (0.00–0.06), respectively. Other negative results for antibody testing included anticardiolipin, voltage-gated calcium channel antibody, chromatin antibody, double-stranded DNA antibody, SCL-70, RNP antibody, Smith, SS-B/SS-A/Ro, and antinuclear antibodies (ANA). The complement C3 and C4 levels were normal. She had a normal erythrocyte sedimentation rate (ESR) in two of her admissions, although C-reactive protein (CRP) was never tested. The patient had a thyroid peroxidase antibody level of 49.1 IU/mL (reference range 0.0–35.0 IU/mL), consistent with hypothyroidism. However, the patient’s known hypothyroidism was well controlled with levothyroxine, as her free T4 was normal and her TSH level remained below 10 uIU/mL (reference range 0.358–3.740).

As stated above, the patient had a history of a pathologic T2 stage A3b small bowel carcinoid, diagnosed 15 years previously. She underwent partial small bowel resection, with no tumor identified in the bowel. She was monitored for remission with 24 h urine tests for 5-hydroxyindoleacetic acid (5-HIAA), serum serotonin, and chromogranin A and showed no disease recurrence. She had no carcinoid symptoms, such as flushing, abdominal pain, or weight loss. We evaluated the patient for carcinoid tumor small bowel recurrence by screening for gastrin, vasoactive intestinal peptide, and 24 h urine 5-hydroxytryptamine levels; all of these were within normal limits.

The patient completed a multitude of broad-spectrum antibiotics and underwent penicillin desensitization due to a penicillin allergy to ensure adequate CSF penetrance, given that her CSF grew Propionibacterium acnes. Following desensitization, she tolerated a course of penicillin. The patient also completed an intravenous immunoglobulin (IVIG) course for possible Guillain–Barré syndrome or chronic inflammatory demyelinating polyradiculoneuropathy. There was no response to either of these treatments. Neurosurgery recommended a leptomeningeal biopsy to assess for the presence of possible malignancy; however, the patient declined this, as it would not have altered her further treatment. Ultimately, the patient and her family opted for comfort measures and pursued hospice care after undergoing multiple tests and treatments and failing to achieve any relief.

## 3. Discussion

Falls can be categorized as arising from two main predisposing and underlying causes: intrinsic factors (related to the individual’s characteristics) and extrinsic factors (related to external environmental conditions) (Table 2). Some of these causes are modifiable and can be adjusted, such as through hearing aids or installing railings and reducing slippery surfaces in the home, while others, such as such as age-related changes in muscle strength or neurological conditions like Parkinson’s disease, are not [1,3]. Sarcopenia, a term commonly used in geriatrics, can fall under either intrinsic or extrinsic factors and is defined as the loss of skeletal muscle mass, which negatively impacts a patient’s quality of life; it may result from age-related changes (primary sarcopenia) or secondary causes such as inactivity, a poor nutritional status, or underlying illnesses [4,5]. Distinguishing between primary and secondary sarcopenia can often be challenging, as both factors may contribute. In the case presented, the patient likely experienced a combination of age-related sarcopenia and the impact of an underlying illness, complicating the distinction between the two.

The differential diagnosis for lower-extremity weakness is broad and complicated by various overlapping conditions, particularly in older patients. Older adults often have aging-related visual, auditory, vestibular, sensory, and motor impairments contributing to falls. However, when evaluating lower-extremity weakness and falls, it is imperative to consider unusual etiologies such as malignancies and infectious and autoimmune disorders. Our patient was a complex case without a clear etiology explaining the progressive weakness in her oculomotor, facial, and lower-extremity musculature despite repeated and extensive laboratory testing and imaging exams.

There are certain autoimmune conditions that can lead to progressively worsening extremity weakness, such as inflammatory myopathies, myasthenia gravis, or Lambert–Eaton syndrome. However, most of these autoimmune conditions tend to affect all limbs equally and symmetrically. The patient in this case had isolated bilateral lower-extremity weakness with the sparing of her upper extremities. She appeared to have been susceptible to autoimmune conditions, given her history of hypothyroidism with elevated thyroid peroxidase positive antibodies of 49.1 and an thyroglobulin antibody level of 208 (levels below 20 IU/mL are typically considered normal). However, studies have shown that the presence of these two antibodies does not necessarily correlate with autoimmune diseases, and elevated levels are seen in normal individuals [6]. Additionally, her thyroxine T4 thyroid hormone and creatine kinase levels remained consistently within normal limits throughout her multiple admissions; therefore, hypothyroid-related myopathy was also deemed unlikely.

Her CSF findings were consistent with hypoglycorrhachia and elevated protein; this is indicative of either chronic inflammation disrupting the blood–brain barrier, an infectious process, or an autoimmune illness such as Guillain–Barré syndrome or chronic inflammatory demyelinating polyradiculoneuropathy (CIDP) [7]. If there was an infectious etiology for the patient’s symptoms, given the chronicity and lack of neutrophil elevation, it would likely have been viral, fungal, or tuberculosis as opposed to bacterial. Further tests, including an autoimmune panel, complement levels, and immunoglobulin levels, were all negative, decreasing the likelihood of her condition being rheumatological in origin. Testing was negative for anti-voltage-gated antibody and anti-acetylcholine receptor antibody, decreasing the likelihood of disorders such as myasthenia gravis, a disorder with a similar presentation.

Carcinoid tumors have been associated with a paraneoplastic disorder involving the peripheral nervous system and can present with peripheral neuropathy and cerebellar dysfunction [8]. Given the patient’s history of a carcinoid and one of her hospital admissions that included symptoms of diarrhea and abdominal pain, the differential diagnosis of either an infectious process or the recurrence of her small bowel neuroendocrine tumor was considered. However, her levels of gastrin, vasoactive intestinal peptide, and 5-hydroxytryptamine from 24 h urine assessing for recurrent carcinoid were all within normal limits. Her abdominal imaging lacked findings suggestive of neuroendocrine tumor recurrence.

Upon reflection on the diagnostic process, it becomes apparent that certain aspects could have been optimized to facilitate the timelier and more precise identification of the underlying cause. A particularly significant oversight was the failure to incorporate a paraneoplastic syndrome into the differential diagnosis, which may have hindered a more focused approach to understanding the patient’s symptoms—especially considering her history of a neuroendocrine tumor. While paraneoplastic syndromes are rare, they can manifest with neurological symptoms, such as peripheral neuropathy, which could have been a pivotal clue in this case. Had this possibility been entertained earlier, particularly during her initial hospitalizations, it might have steered the diagnostic trajectory and treatment strategy in a more targeted direction.

Additionally, the absence of electromyography (EMG) and nerve conduction studies (NCS)—both not readily available in the inpatient setting and uncertain in terms of outpatient application—represents another lapse in diagnostic testing. These tests could have offered crucial insights into the motor and sensory disturbances that the patient experienced, potentially indicating whether the symptoms stemmed from a primary neuropathic process or an alternative underlying condition. We aim to highlight that recognizing these gaps in the diagnostic approach is crucial for clinicians managing similarly complex cases. By giving earlier consideration to these diagnostic tools and possibilities, a more accurate diagnosis could have been reached, potentially accelerating the path to a more effective and timely treatment plan.

In investigating the possibility of an infectious etiology, tuberculosis testing was performed. She had a positive IGRA test and Fungitell assay but lacked other findings suggestive of tuberculosis. Her chest X-ray showed no acute or chronic infiltrates. Additionally, CSF culture and PCR were negative for tuberculosis. The elevated beta 1,3-D-glucan, resulting in a positive IGRA test, was possibly related to the multiple antibiotics administered during her treatment. Many antimicrobial drugs cause the elevation of beta 1,3-D-glucan, given the reactivity between the assay used to test for beta 1,3-D-glucan and certain antibiotics [8]. Therefore, a fungal infection was unlikely.

Her CSF Lyme serology was considered positive, and, despite completing 2 weeks of ampicillin and intravenous penicillin, she failed to show a clinical improvement. She was also treated for presumed Bell’s palsy with antiviral medications and steroids. Several viral agents are suggested in the etiology of Bell’s palsy, specifically reactivated viruses such as varicella zoster virus (VZV) and herpes simplex virus type 1 and type 2 [9]. In this case, however, Bell’s palsy was deemed unlikely, given that the CSF PCR was negative for infectious agents associated with Bell’s palsy.

Moreover, in differential diagnosis, one should consider chronic inflammatory demyelinating polyneuropathy (CIDP). Its onset is often preceded by a respiratory or gastrointestinal infection, triggering a T-cell-mediated autoimmune attack against the peripheral nerve and root myelin. However, increased white blood cells in CSF are not typical, and a CIDP diagnosis is less likely with a higher number of leukocytes [10]. An infectious etiology was considered and treated appropriately without success. Clinically, she did not present as a traditional CIDP patient, given that her weakness spared her upper extremities.

The patient’s stool testing suggested a possible campylobacter infection, and, with the associated gastroenterology symptoms, Guillain–Barré syndrome (AIDP) was considered in the differential diagnosis. This disorder is due to the autoimmune correlation with this type of bacterial infection [11]. GBS treatment was initiated with IVIG. Additionally, her spinal fluid culture grew Propionibacterium acnes, and, as a result, the patient received 2 weeks of ampicillin but failed to show any improvement despite multiple antibiotic agents and IVIG.

Although transverse myelitis (TM) was, at one point, considered for a differential diagnosis, there were not enough objective data substantiating this diagnosis. Transverse myelitis is often misdiagnosed as GBS because of the similarities in sensory loss and weakness; however, TM is associated with symptoms corresponding to a spinal cord level and typically involves symmetrical paralysis and/or paresthesia [11]. Our patient had asymmetric syndromes with waxing and waning levels of strength; therefore, TM was an unlikely etiology for our patient’s symptoms.

The differential diagnosis for her paresis was subsequently narrowed down to carcinomatosis meningoencephalitis. This is a rare disorder whereby cancerous cells from a primary source, such as the mammary gland or gastrointestinal tract, seed into the leptomeninges [12]. The patient had a history of a neuroendocrine tumor of the small bowel, and, although it was in remission, it still rendered her more susceptible to carcinomatosis meningoencephalitis [12]. Additionally, her CSF lumbar puncture results showed hypoglycorrhachia and elevated protein, and, given that infectious causes were ruled out, carcinomatosis meningoencephalitis was considered a likely diagnosis [12]. In most cases, the best initial diagnostic modality is MRI with gadolinium, as this test is both highly sensitive and specific; however, inconclusive cases can be further evaluated with a biopsy [13]. This condition has a poor prognosis.

The patient was presented with the option of a leptomeningeal biopsy by interventional neurology to further investigate the underlying cause of her symptoms. However, after thorough consideration, it was determined that the biopsy would likely have a negligible impact on her prognosis. While we acknowledge the inherent limitation of not obtaining a definitive diagnosis through biopsy, our intent is to shed light on the diagnostic journey—emphasizing the critical need to consider rare and complex causes of symptoms, rather than defaulting to the assumption that they are merely age-related. Given the advanced stage of her illness and the generally poor prognosis associated with carcinomatosis meningoencephalitis, it was felt that the biopsy would not yield meaningful benefits in terms of treatment or survival outcomes. In alignment with the patient’s wishes, and after extensive discussions with her family and healthcare team, the decision was made to transition to palliative care, prioritizing comfort and quality of life during this profoundly challenging time as per the patient’s request.

## 4. Conclusions

This case highlights the critical importance of a comprehensive and systematic approach to diagnosing bilateral lower-extremity weakness and falls, particularly in older adults, where differential diagnosis can be influenced by assumptions about age-related decline. By broadening the diagnostic lens to include rare and less obvious neurological conditions, clinicians can avoid oversimplifying complex presentations and provide more accurate, tailored care so as to avoid rehospitalizations. This case also emphasizes the value of a patient-centered approach, ensuring that the treatment decisions align with the patient’s wishes, particularly when curative options are no longer viable. Ultimately, it serves as a reminder to healthcare providers to stay vigilant, adaptable, and compassionate, considering all possibilities in the pursuit of a diagnosis and effective care.

## Figures and Tables

**Table 1:** Lumbar puncture results. The table illustrates the results of the patient’s lumbar puncture and the resulting information found from the CSF analysis.

Lumbar Puncture Results			
	Third Lumbar Puncture	Second Lumbar Puncture	Initial First Lumbar Puncture
CSF Appearance and Color	Cloudy, colorless	Yellow cloudy/hazy	Xanthochromia
CSF WBC	434	163	157
CSF RBC	3	29	18
CSF Glucose	46	19 mg/dL	6
CSF Total Protein	366	>460 mg/dL	460

**Table 2:** (Adapted from references [1,3]). The causes of falls can be broadly categorized into intrinsic and extrinsic factors. Extrinsic factors stem from the environment, while intrinsic factors are inherent characteristics of the patient. Extrinsic factors are typically modifiable, meaning that they can be adjusted or altered to reduce the risk of falls.

Etiology of Falls	
Intrinsic	Extrinsic
Age-related physiological changes (e.g., sight and hearing impairments, balance issues)	Environmental hazards (e.g., obstacles, slippery surfaces, poor lighting)
Pathological conditions (e.g., neurological, cardiovascular, endocrine, psychiatric disorders)	Inadequate footwear or attire (e.g., improper fit, lack of support)
Musculoskeletal issues (e.g., muscle weakness, reduced range of motion)	Exposure to extreme temperatures can lead to dizziness or weakness, contributing to falls
Age-related physiological changes (e.g., presbyopia and hearing impairments, balance issues)	Alcohol consumption or use of sedative medications that can impair coordination and balance (Beers Criteria)

## Data Availability

Data supporting the reported results are available upon reasonable request. Ethical and privacy constraints prevent full data disclosure.
